# Overcoming the impact of physiologic tremors in ophthalmology

**DOI:** 10.1007/s00417-022-05718-2

**Published:** 2022-07-05

**Authors:** Gurfarmaan Singh, Wilson Wong Jun Jie, Michelle Tian Sun, Robert Casson, Dinesh Selva, WengOnn Chan

**Affiliations:** 1 School of Medicine, University of Adelaide, Health & Medical Sciences Building, 4 North Terrace, Adelaide, SA 5000 Australia; 2 Royal Adelaide Hospital, Adelaide, SA Australia

**Keywords:** Physiologic tremors, Ophthalmology, Robotics, Instruments, Exoskeletons, Technique modifications, Lifestyle factors

## Abstract

**Purpose:**

Ophthalmic surgery involves the manipulation of micron-level sized structures such as the internal limiting membrane where tactile sensation is practically absent. All humans have physiologic tremors that are of low amplitude and not discernible to the naked eye; they do not adversely affect the majority of the population’s daily functioning. However, during microsurgery, such tremors can be problematic. In this review, we focus on the impact of physiological tremors on ophthalmic microsurgery and offer a comparative discussion on the impact of such tremors on other surgical specialties.

**Methods:**

A single investigator used the MEDLINE database (via PubMed) to search for and identify articles for inclusion in this systematic review. Ten key factors were identified as potentially having an impact on tremor amplitude: beta-blockers, muscle fatigue, robotic systems, handheld tools/micromanipulators, armrests/wrist supports, caffeine, diet, sleep deprivation, consuming alcohol, and workouts (exercise). These key terms were then searched using the advanced Boolean search tool and operators (i.e., AND, OR) available on PubMed: (*keyword*) *AND* (surgeon tremor *OR* microsurgery tremor *OR* hand steadiness *OR* simulator score).

**Results:**

Ten studies attempted to quantify the baseline severity of operator physiologic tremor. Approximately 89% of studies accessing the impact of tremors on performance in regards to surgical metrics reported an improvement in performance compared to 57% of studies concluding that tremor elimination was of benefit when considering procedural outcomes.

**Conclusions:**

Robotic technology, new instruments, exoskeletons, technique modifications, and lifestyle factors have all demonstrated the potential to assist in overcoming tremors in ophthalmology.

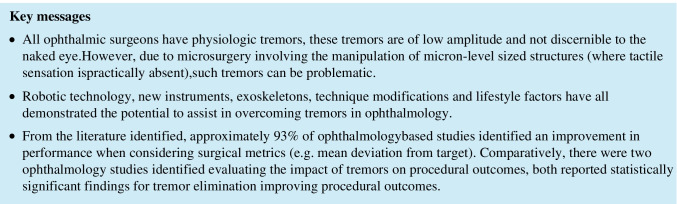

**Supplementary Information:**

The online version contains supplementary material available at 10.1007/s00417-022-05718-2.

## Introduction

Ophthalmic surgery involves the manipulation of micron-level sized structures such as the internal limiting membrane where tactile sensation are practically absent and subsequently the procedure is performed solely on the basis of visual feedback [1]. Negotiating such microsurgeries understandably requires a great deal of precision and maybe limited by a surgeon’s physiological tremor.

A tremor is defined as a rhythmic and involuntary movement of any body part and is the most common movement disorder [2]. Tremors can broadly be divided into two main categories: physiological and pathological. Physiological tremor are bilateral low-amplitude tremors with an average frequency of 7.7 Hz [3, 4]. All humans have a physiologic tremor, which may be unnoticed unless testing is conducted [5]. As physiological tremors are of low amplitude and not discernible to the naked eye, they do not adversely affect the majority of the human population’s daily functioning or cause limitation in their activities of daily living. However, during microsurgery, such tremors can be problematic. An accentuated physiologic tremor is differentiated from a pathological tremor on clinical grounds (i.e., is visible) and in the presence of magnifying factors. Stressors that may magnify physiological tremors include drug-induced (e.g., caffeine or anti-depressants), metabolic (e.g., hyperthyroidism and hypoglycemia), and anxiety [6].

Physiological tremors can be accentuated by postural movements and muscle contraction [7]. Lakie et al. found that large changes in posture had no effect on severity of tremor,however, slow wrist flexion and extension maneuvers significantly worsened the magnitude of tremor recorded [8]. In the case of contraction tremors, one study concluded isometric and tonic contraction both result in worsening of an individual’s physiologic tremor [9]. Another factor affecting the severity of one’s physiologic tremor is fatigue. Chandra et al. reported that increasing fatigue over time resulted in an increase in hand tremor during simulated laparoscopic surgery tasks [10]. Based on these findings, postural movements, muscle contraction (isometric and tonic), and fatigue may be key modifiable factors to reduce the impact of tremors in ophthalmology. In this review, we focus on the impact of physiological tremors on ophthalmic microsurgery and offer a comparative discussion on the impact of such tremors on other surgical specialties that employ microsurgeries. Secondly, this review aims to summarize the many strategies employed to overcome these tremors and make ophthalmic microsurgery more efficient and safer.

## Methods

### Protocol

The PRISMA (Preferred Reporting Items for Systematic Re-views and Meta-Analyses) criterion was used as the structural basis for this systematic review. The review was aimed at identifying existing literature on the impact of tremor on ophthalmic surgery and to explore the evidence surrounding various modalities for overcoming tremors in ophthalmology.

### Search strategy

A single investigator used the MEDLINE database (via PubMed) to search for and identify articles for inclusion in this systematic review. Additionally, the reference lists from identified articles were reviewed and relevant articles were included if they were deemed appropriate. Through an initial literature search, 10 key factors were identified as potentially having an impact on tremor amplitude: beta-blockers, muscle fatigue, robotic systems, handheld tools/micromanipulators, armrests/wrist supports, caffeine, diet, sleep deprivation, consuming alcohol, and workouts (exercise). These key terms were then searched using the advanced Boolean search tool and operators (i.e., AND, OR) available on PubMed: (*keyword*) *AND* (surgeon tremor *OR* microsurgery tremor *OR* hand steadiness *OR* simulator score). There were no date restrictions applied to the database searches, with all articles published until June 15, 2020 being considered. Only articles published in English were included in the review.

### Study selection

**Figure 1:**
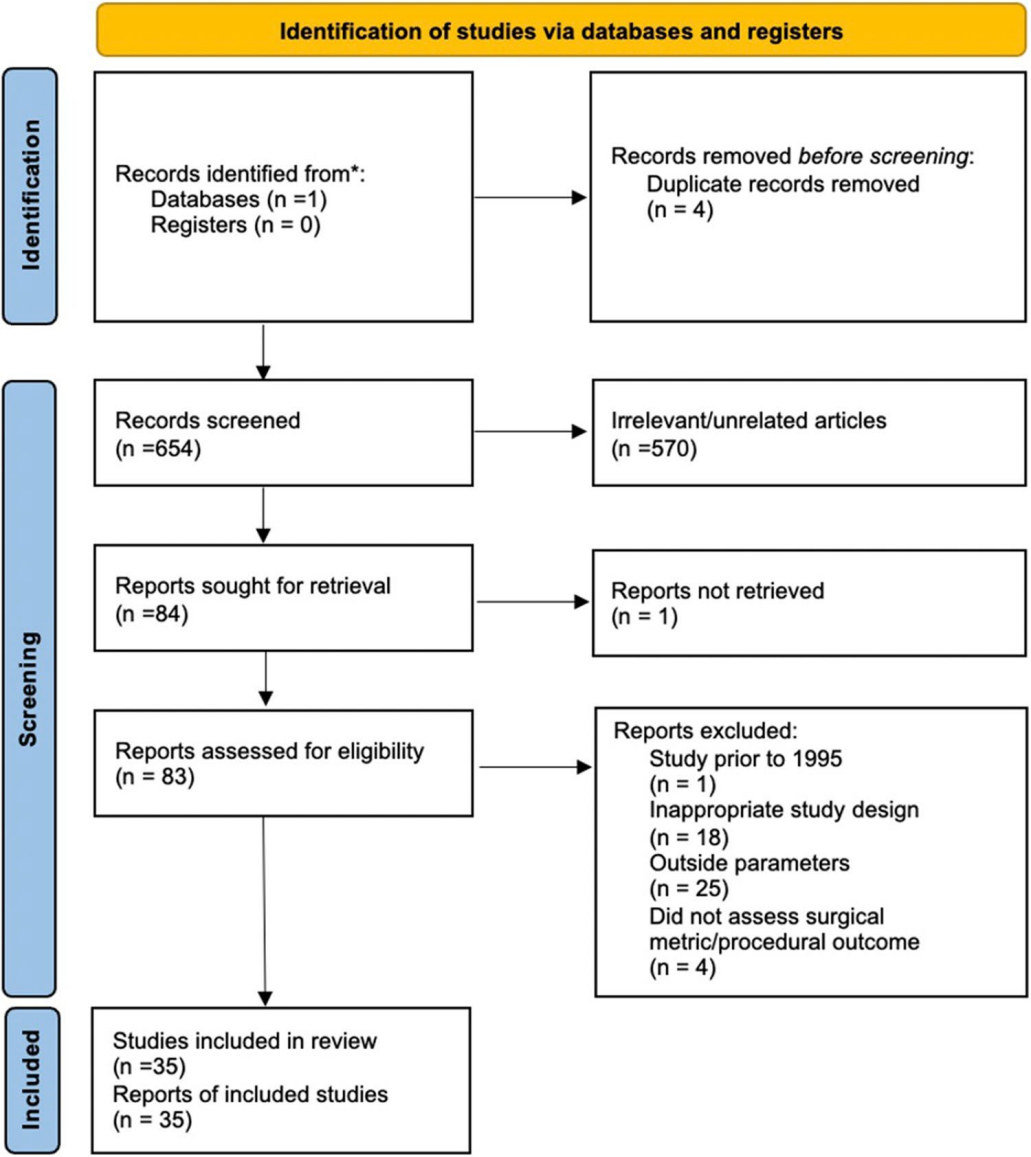
PRISMA flow diagram for identification of studies [11]

Through the “save” option, each keyword’s search results were downloaded in the .CSV format. A manual assessment of the titles and abstracts from these article lists were then used to filter for relevant articles and remove all duplicate articles identified. All articles deemed relevant were subsequently transferred to a separate Microsoft Excel Spreadsheet. This process generated a database consisting of 84 articles that either evaluated the impact of the selected factors on tremors and/or on surgical performance being selected in the first phase of screening. The final stage of screening involved accessing the full texts of all the 83 articles in order to identify studies measuring the impact of tremors on surgical metrics and/or procedural outcomes which resulted in 35 articles being identified (see Figure 1 for PRISMA flowchart for study identification).

### Eligibility criteria

The final stage of screening selected articles based on the following criterion: (1) articles must be laboratory studies, prospective studies, animal studies or retrospective studies; (2) must discuss or suggest one of the ten key factors mentioned above as having an implication on tremor and the outcome being accessed; and (3) outcomes can be in the form of surgical metrics (e.g., accuracy, error rate, time to completion) or procedural outcomes (e.g., successful completion rate or post-procedure complications). The only exclusion criteria were if articles were review based (i.e., comprehensive reviews, systematic reviews, and meta-analyses), case reports or patient/animal series, and if they were published prior to 1995. All studies with medical students, resident surgeons, consultant surgeons, and non-surgeon participants were included.

### Data extracted

For this review, every study deemed to fit the criteria for inclusion had the following data extracted: title of publications, authors and affiliations, country of study, year of study, strategy to overcome impact of tremors, surgical metric or procedural outcome accessed, impact on metric or outcome, specialty, and number of operating participants.

The findings of this review are presented in narrative form, as no data was found to be appropriate for pooling for statistical analysis. Furthermore, due to the nature of the studies (limited sample sizes) and the considerable variation in assessed outcomes (several studies considering subjective outcomes), there likely is a degree of publication bias to consider.

### Assessment of study evidence

All of the selected articles were then accessed by the reviewer (G. S.) and assigned a level of evidence according to The Oxford 2011 Levels of Evidence (OCEBM) [12]. The assigned grades were than checked by a second reviewer (W. C.) and any discrepancies were discussed in order to arrive at a final grade.

## Results

From the final data set 10 studies attempted to quantify the baseline severity of operator physiologic tremor (Table 1). Techniques to gauge the severity of tremor included subjective severity while undertaking surgical procedures, ability to maintain a specific offset distance from a target, and deviation from a stationary target when attempting to maintain position for a set time. As seen in Table 1 there was considerable variability in the methods of assessing baseline tremor and the units of measurement of baseline severity. Therefore, it was not deemed feasible or reliable to calculate a mean baseline tremor (e.g., in Hz).

**Table 1:** Studies reporting baseline tremor severity during surgical procedures and/or simulation tasks

Author	Method/unit of tremor measurement	Score
Feng et al. [13]	Subjective microvascular tremor scale	N: 2.4, E: 0.9*
Nakano et al. [14]	Average pointing error + maximum error (μm)	A: 70, M: >300
Yang et al. [15]	RMS for pointing task (μm)	112
Song et al. [16]	RMS for drift from a defined offset height (μm)	L: 43.4, S: 36.0
Maclachlan et al. [17]	3D maximum error (μm)	N: 264, E: 318
Song et al. [18]	Tool tip motion towards target (μm)	1000
Zhang et al. [19]	Average deviation of tool tip (μm)	23.7
Yang et al. [20]	RMSE for holding tip still (μm)	90
	Maximum error for holding tip still (μm)	240
Okamura et al. [21]	Mean tremor amplitude without FMA (pix)	699.4
	Subjective tremor score (pts)	3
Csókay et al. [22]	Instrument tip movements (mm)	0.6

Table 1 aims to highlight the significant differences in method of tremor severity measurement within included studies. *RMS* root mean square, *RMSE* root-mean-square error, *N* novice operator, *E* expert operator, *L* longer time, *S* shorter time, *A* average, *M* maximum, *μm* micrometers, *pts* points, *pix* pixels

**Figure 2:**
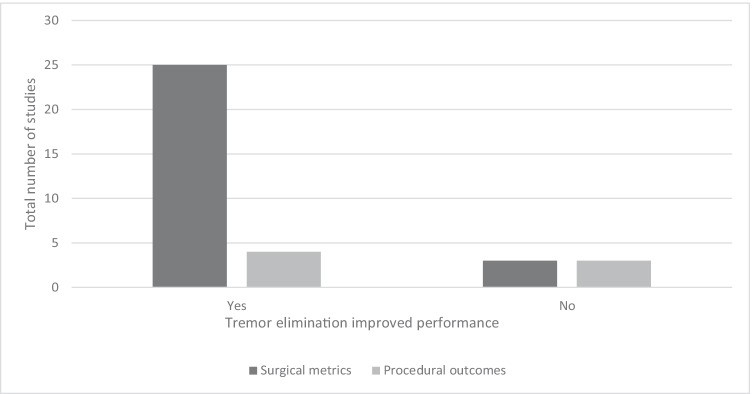
Comparison of studies evaluating if tremor elimination improved performance when considering surgical metrics vs. procedural outcomes

Figure 2 highlights that approximately 89% of studies accessing the impact of tremors on performance in regards to surgical metrics reported an improvement in performance upon reduction and/or elimination of operator physiological tremor. As seen in Table 2, there were 12 ophthalmology specific studies identified, of which 11 found an improvement in performance when eliminating tremors. Two studies employed medications for tremor reduction, one used a robotic surgical system, and nine studies evaluated surgical metric performance when a handheld tremor reduction tool was used.

**Table 2:** Ophthalmology-based studies reporting the impact of physiologic tremors on surgical metrics

Author	Strategy to reduce tremors	Surgical metric	Improved performance	Speciality	Evidence quality	Year	Country	***n***
Roizenblatt et al. [23]	Beta-blocker (tremor reducing), caffeine (tremor increasing)	Simulator score	Yes (beta-blocker)	Ophthalmology	II	2020	Brazil	15
Pointdujoir et al. [24]	Beta-blocker (propranolol)	Simulator score	Yes	Ophthalmology	III	2011	America	18
Nakano et al. [14]	Robotic surgical system	Stability and accuracy	Yes	Ophthalmology	IV	2009	Japan	NS
Yang et al. [15]	“Micron” handheld robotic micromanipulator	Physiologic tremor filtration and RMS error	Yes	Ophthalmology	IV	2015	America	1
Song et al. [16]	Swept source optical coherence tomography-based “SMART” handheld tool	Maintained tool tip offset distance	Yes	Ophthalmology	IV	2012	America	2
Maclachlan et al. [17]	Actively stabilized handheld tool	Position error on several error metrics	Yes	Ophthalmology	IV	2013	America	6
Gonenc et al. [25]	“Micron” handheld robotic micromanipulator	Magnitude of forces during membrane peeling of a phantom bandage and membrane on raw chicken egg	Yes	Ophthalmology	IV	2012	America	1
Song et al. [18]	Fibre-optic OCT sensor-guided SMART microforceps	Targeted grasping and peeling performance	Yes	Ophthalmology	IV	2013	America	NS
Zhang et al. [19]	Hand-held instrument with active tremor compensation	Tip force applied while peeling inner shell membrane of chicken egg	Yes	Ophthalmology	IV	2020	China	1
Gonenc et al. [26]	Micron with force sensing	Peeling force and peeling speed on phantom bandages and eggs	Yes	Ophthalmology	IV	2014	America	1
Yang et al. [20]	Micron with force sensing	RMS error for holding-still and circle tracing in eye phantom and rubber target	Yes	Ophthalmology	IV	2013	America	1
Okamura et al. [21]	Freely movable arm rest (FMA)	Arm fatigue, subjective tremor score, and suturing accuracy	No	Ophthalmology	IV	2020	Japan	8

*RMS* root mean square, *RMSE* root-mean-square error, *N* novice, *E* expert, *L* longer time, *S* shorter time, *A* average, *M* maximum, *MTS* microvascular tremor scale, *TTC* time to completion, *TLM* transoral laser microsurgery, *RVBS* retinal vein bypass surgery, *NS* not specified

Comparatively, there were only 16 studies identified from other surgical specialities within medicine; approximately 81% of these studies reported an improvement in surgical metric performance when a tremor eliminating strategy was employed (See Online Resource 1) [10, 17, 27–40]. The literature evaluating the impact of tremor elimination on procedural outcomes was far scarcer, with only seven studies identified (Table 3). Overall, only 4/7 (approximately 57%) of studies concluded that tremor elimination was of benefit when considering procedural outcomes. The two ophthalmology studies identified both employed the use of surgical robotic systems to eliminate tremors and both reported statistically significant findings for tremor elimination improving procedural outcomes.

**Table 3:** Studies evaluating procedural outcomes

Author	Strategy	utcome	Improved performance	Specialty	Evidence	Year	Country	*n*
Bernie et al. [41]	Da Vinci surgical system	TTC, blood loss, hospital stay, complications	Yes (hospital stay + number of complications)	Urology	IV	2005	America	1
Chen et al. [42]	Robotic surgical system	RVBS success rate in ex vivo porcine eyes	Yes	Ophthalmology	IV	2017	China	1
Knight et al. [43]	Zeus surgical system	Patency of rat femoral artery anastomoses	No	Pediatric surgery	IV	2005	America	NS
You et al. [44]	Da Vinci surgical system	TTC, hospital stay, complications, and mortality	No	General surgery	III	2013	Korea	1
Fleming et al. [45]	Co-op robotic surgical assistant	Time to cannulation, successful cannulation, and successfully maintained cannulation	Yes	Ophthalmology	IV	2008	America	19
Csókay et al. [22]	I–III finger support, which holds the instruments on Bethlehem bridge	Complication rate from 23 cases	Yes	Neurosurgery	III	2009	Hungary	NS
Basaran et al. [40]	Fatigue and sleep deprivation	Anastomosis patency rates	No (less fatigue)	Plastic surgery	III	2015	Turkey	1

*RMS* root mean square, *RMSE* root-mean-square error, *N* novice, *E* expert, *L* longer time, *S* shorter time, *A* average, *M* maximum, *MTS* microvascular tremor scale, *TTC* time to completion, *TLM* transoral laser microsurgery, *RVBS* retinal vein bypass surgery, *NS* not specified

## Discussion

Overall, our findings tend to support the notion that physiologic tremor reduction has a positive impact on surgical performance when considering surgical metrics or procedural outcomes across many specialties, including ophthalmology in particular.

Two studies from Table 1 (evaluating operator baseline tremor) differentiated between the baseline tremors of novices and expert participants. Feng et al. noted that the mean tremor score of their microvascular naïve precipitants was 2.40 during freehand completion of tasks (statically significant). However, their single microvascular expert operator had a score of 0.86; this was not statically significant given a sample size of 1 [13]. Interestingly, Machlachlan et al.’s study noted that the 3D maximum error was higher in surgeon participants compared to their less experienced counterparts at baseline/without tremor eliminating intervention (264 μm versus 318 μm, respectively). External to the study, one study comparing the performance of residents to their more experienced consultant anterior segment colleagues noted that the specialist cohort had superior anti-tremor simulator scores [46]. Overall, data comparing the baseline physiologic tremors of expert ophthalmic surgeons to novices is very limited. The more robust data tends to suggest that novice surgeons have a greater baseline physiologic tremor than their more experienced counterparts. This may be attributed to greater time spent training (therefore more repetition), resulting in less stress and greater familiarity with specific maneuvers intraprocedurally.

### Medications

A potential solution to overcoming tremors in ophthalmology may be through the use of medications. Propranolol is commonly used for managing hypertension, cardiac arrhythmias, myocardial infarction, migraine, portal hypertension, anxiety, essential tremors, and hyperthyroidism [47]. Elman et al. discovered a dose prior to operating reduced the tremors of ophthalmology residents [48]. Similarly, another study demonstrated that propranolol resulted in a 22% decrease in the magnitude of the tremors of ophthalmologists [49]. Lubahn et al. concluded that another beta antagonist (timolol) was not effective in alleviating hand tremors [50]. Arnold et al. also reported that timolol was only effective in reducing caffeine-induced tremors of ophthalmology trainees [51]. An additional consideration to the use of beta-blockers for tremor control is the potential for side effects. There was a total of seven beta-blocker studies identified in this review and only three had discussion on adverse effects. Arnold et al. reported two (out of 16 participants) withdrew due to unspecified side effects, whereas two other studies stated no participants experienced any adverse effects [48, 49, 51]. These findings, in addition to 2/3 studies reporting that a beta-blocker was effective in improving surgical performance through tremor reduction, allude to select beta-blockers being effective in reducing tremors and subsequently improving intraoperative performance. Presently data is extremely limited and larger, more robust studies are needed to confirm if this reduction in tremors offers improved surgical performance without side effects.

### Fatigue

Ophthalmology surgeries can often be completed in less than 30 min [52]. During this time a high degree of concentration is required and this over the course of a day can result in increasing fatigue. The onset of physical fatigue over the course of a day can result in the increase in amplitude of a surgeon’s physiological tremor. One study demonstrated that muscle-cooling was effective in reducing the amplitude of fatigue-induced tremors [27]. The trial demonstrated a statistically significant reduction in tremor amplitude of experienced surgeons when wearing a muscle cooling suit (5 °C). The trial failed to show statistical significance for trainees. If larger studies are conducted and the results continue to be promising, muscle-cooling suits may have a role to play in the reduction of ophthalmologists’ tremors. One key limitation to consider is the design of the cooling suit itself, the physical specifications of the suit may impair intrinsic hand dexterity and thereby limit its use in microsurgery.

Sleep plays a vital role in maintaining optimal functioning of the human body. It is recommended that individuals between the ages 18 and 60 obtain at least 7 h of sleep each night [53]. Presently, no literature exists that quantifies the impact of sleep on an ophthalmologist’s physiologic tremor. A study of ophthalmology residents did however conclude that acute sleep deprivation did not have an impact on overall performance of a simulated anterior segment surgical task [54]. Basaran et al.’s findings suggest that there was a decrease in anastomosis success when comparing the procedures done in the morning versus those done at the end of the day (fatigued) or the following morning without sleeping, however, their results did not demonstrate statistical significance [40]. They did however report a statistically significant reduction in error score, autopsy score, and global rating score when comparing the evening and morning groups. These findings suggest that sleep may play a role in decreasing surgical performance. In contrast, several studies not included in the final data have concluded that sleep deprivation did not demonstrate a detectable decrease in surgical performance [55–57]. Based on the data available it is difficult to ascertain if sleep deprivation has a significant impact on the physiological tremors and surgical performance of ophthalmologists.

### Robotic surgical systems

Recent times have seen increasing research and attempts to incorporate robotics into ophthalmic surgery in order to improve patient outcomes [58]. This study identified a total of 14 studies (10 evaluating surgical metrics and 4 evaluating outcomes) assessing the impact of tremor on surgical performance through the use of robotic surgical systems. Approximately 65% of the studies concluded that there was a benefit to surgical performance. Three of the studies were noted to state that there was no benefit to performance through the use of tremor filtering robotic systems. Interestingly, a fourth study by Prasad et al. suggested that the motion scaling, rather than tremor cancellation offered by robotic surgical systems, improves surgical accuracy.

Only two of the robotic surgery studies identified were ophthalmology based, both of which evaluated or used their self-designed systems. External to the review, case reports have been published of the da Vinci system being successfully used to perform used for pterygium surgery, penetrating keratoplasty, and amniotic membrane transplant surgery in humans [59–61].

From the review of literature, seven studies evaluating the impact of tremor elimination on surgical outcomes were deemed appropriate for inclusion (see Table 3). Notably, five of these studies employed the use of robotic surgical systems.

In 2005, Knight et al. compared the use of the tremor eliminating Zeus Surgical System to traditional freehand femoral artery anastomosis in rats [43]. They reported that the robotic system offered no measurable benefit when considering surgical outcomes, including success of femoral artery anastomosis patency or leakage rates after 3 min. In a more ophthalmology specific context, Fleming et al. evaluated outcomes of a robotic system being employed to cannulate chorioallantoic membranes of chicken embryos (imitating retinal microsurgery) [45]. They concluded with learning time that the robotic system improved both maintenance of cannulation and time for successful cannulation. Similarly, Chen et al. compared the efficacy of a custom-made retinal microsurgery robotic system to the conventional free-hand approach, assessed via the successful completion of retinal vein bypass surgery (RVBS), using porcine eyes [42]. They found that the tremor eliminating system significantly improved RVBS success rates compared to the free-hand approach (35% vs. 5%, respectively). While this data is very limited, tremor elimination using robotic systems do appear to be beneficial in enhancing surgical outcomes.

Literature on the efficacy of tremor eliminating robotic systems on procedural outcomes in human subjects is also similarly scarce. Bernie et al. found that the patients who underwent robotic pyeloplasty had lower post-operative complications and length of hospital stay compared to the traditional approach [41]. In contrast to this, You et al. found that the Da Vinci Surgical System was relatively equivocal to laparoscopic adrenalectomy when considering surgical outcomes such as completion time, complications, mortality, and length of hospital stay [44].

The Intraocular Robotic Interventional Surgical System (IRISS) is another robotic surgical system currently under development; unlike the da Vinci it is specifically designed for use in ophthalmology. Wilson et al. created the IRISS to be employed as both a master-slave manipulator and a fully automated robot. The IRISS was demonstrated to be able to successfully perform a range of ocular procedures including vitrectomies, retinal-vein cannulation, and lens aspiration on pig porcine eyes [62]. Chen et al. also used the IRISS to perform semi-automated OCT-guided cataract surgeries,their system successfully achieved complete extraction in over 80% of pig eyes and in the remaining eyes almost complete extraction was achieved, with no reported capsule ruptures [63]. The IRISS has thus far shown great promise in successfully performing several ophthalmic procedures on pig porcine eyes,with further development its widespread incorporation may play a vital role in future ophthalmic practice and elimination of challenges associated with operator physiologic tremor.

Another robotic system showing a great deal of promise is the Steady Hand Eye Robot 2 (SHER2), and similarly to the IRISS, it is specifically designed for ophthalmic microsurgery. The robot actuates and mimics the operator’s movements with the robotic arm and filters out any detected tremors. Gonenc et al. demonstrated that the SHER tool reduced oscillations of 2–15 Hz in magnitude by over 50% while peeling the inner shell membrane (ISM) of chicken embryos [64]. Data on the SHER2 is currently limited, however with further research and development it may be the solution to addressing the challenges associated with a surgeon’s physiological tremor.

### Robotic devices

This review of literature identified a total of 10 articles discussing the use of tremor reducing micromanipulators and their impact on surgical performance. Six of the studies assessed a micromanipulation handheld tool known as the “Micron” with/without additional modifications to assist retinal surgeons [15, 17, 20, 25, 26, 36]. The development of the Micron commenced in 1996 and Riviere et al. published the earliest literature on a Micron prototype impacting operator tremor 7 years later [65, 66]. They described the Micron’s handle being separated from the tip by piezoelectric actuators, in doing so allowing for the tip to move independently to the handle. The earliest version of the Micron identified in this study was published by Choi et al. in 2007 [36]. In this version an optical sensing system and orthogonal accelerometers were used to provide feedback and allow the tip to adjust for deviation from target point due to tremor. Several subsequent studies have been published incorporating additional technology such as a force-sensing needle tip in order to further improve the Micron instrument [20, 26]. Notably, Yang et al. reported that the Micron resulted in an approximately 90% reduction in physiologic tremor during a pointing task and error less than 25 μm during circle tracing. They concluded that this reduction in error was well within parameters for safe laser photocoagulation in Diabetic Retinopathy patients [15]. The results of the remaining five studies assessing the use of the Micron also demonstrated an improvement in surgical metric performance as a result of the tremor reduction offered by the use of the micromanipulator. While present literature is promising, it is important to note that all of the studies are based on phantom models or animal studies and more robust safety studies will be required prior to its realistic implementation into ophthalmic practice.

Similarly, Song et al. developed a “SMART” (Smart Micromanipulation Aided Robotic-surgery Tool) tool to assist vitreoretinal surgeons [16]. Their results also demonstrated that surgeon hand tremor was reduced with the use of the SMART tool. From a surgical metric point of view, its use increased tool stability, thereby helping maintain a constant stand-off height from the chick embryo’s membrane. In the following year, Song et al. also developed SMART OCT-guided microforceps for microsurgery, which was also reported to improve surgical performance by providing superior grasping and peeling compared to freehand use [18]. The incorporation of robot master-slave systems may make such devices obsolete in leading centers, however, these tools are a considerably more economical alternative in less wealthy geographic regions.

In vitreoretinal surgery, closure of the forceps during membrane peeling in order to manipulate retinal structures requires delicate precision and controlled movements [36]. The presence of a physiologic tremor can interfere with efficient completion of this process. Romano et al. reported the use of foot controllers for closure of pneumatic forceps rather than conventional manual hand-controlled closure [67]. The use of a foot controller eliminates the impact of physiologic tremors on the ability of a surgeon to grasp and manipulate a patient’s retinal membrane. The initial results are promising, with no retinal breaks found intraoperatively or post-operatively.

Chan et al. developed a foot-controlled endoscope holder to assist with sinus surgery [68]. The device involves the use of a foot control which consists of an inertial measurement unit (IMU) and Bluetooth capabilities to connect to the robotic arm. The robot arm consists of four points of joint movement. Inversion and eversion of the foot allow the surgeon to navigate through each joint and select the specific joint they would like to move. Raising their heel off the ground and moving it left or right allows the specific joint selected to be manipulated. This extra arm coupled with the surgeon’s two hands produce a three-hand technique, theoretically offering a great deal of precision and possible tremor reduction. This design is currently in its developmental stage and data is not yet available to determine how significant of a tremor reduction this additional hand may offer.

### Exoskeletons

The review of literature conducted did not identify any current exoskeletons being employed to overcome the impact of tremor on microsurgery; however, their potential in reducing the severity of pathological tremors presents a possibility of their future use to bolster ophthalmic surgeon performance. An example of the exoskeleton is the wearable orthosis for tremor assessment and suppression (WOTAS), which showed a 40% tremor reduction for all 10 participants with pathologic tremors [69]. In its active mode (greatest reduction in tremor), the WOTAS generates forces that directly oppose the direction of the tremor, thereby cancelling out motion. Gallego et al. demonstrated a neuroprosthesis that was effective in reducing the amplitude of tremor by an average of 52% [70]. The neuroprosthesis delivers an injecting current via transcutaneous patches to trigger an antagonistic contraction of muscles, subsequently cancelling the motion of the tremor. Though presently in its infancy, these exoskeletons may play a key role in overcoming tremors in ophthalmology. They may be particularly beneficial in areas where it is not possible to use a da Vinci system (e.g., financial constraints in developing countries). Much like the cooling suits, the design of the exoskeletons may limit the dexterity of ophthalmologists and subsequently prevent its incorporation into ophthalmic microsurgery.

### Ergonomic modifications

The alteration of instrument grip, finger configuration, and the use of orthotic supports have been demonstrated to reduce the impact of tremor during microsurgery. Csókay et al. describe a microsurgical technique which involves the use of 1st–3rd fingertip method with the addition of a Bethlehem bridge (support orthosis) during microneurosurgery [22]. Their comparison of 23 human cases with and without the technique demonstrated a reduction in complications (i.e., improvement in surgical outcomes). Another study reported that robotic arm holder known as the “EXPERT” decreased surgeon fatigue and decreased difficulty in performing microneurosurgical procedures [39]. In the 3 years prior to Goto et al.’s publication, the EXPERT was used in 13 procedures on live patients and demonstrated no associated complications or mortality. In contrast to this, the previously discussed article by Okamura et al. found that their FMA (freely moveable armrest) did not improve performance through reduction of hand tremor [21].

Anecdotally, resting the hand on the brow with a superior approach is also believed helpful for tremor reduction during ophthalmic surgery. Prior to the 1990s the superior approach was the approach of choice for cataract surgeries. In the early 1990s Dr I. Fine described a clear corneal incision with a temporal approach [71]. This approach likely concedes a significant advantage of tremor reduction due to wrist support but offers greater technical benefit including improved ability for the patient to fixate on the microscope light due to an unobstructed visual axis, improved exposure to the surgical limbus, and being more astigmatically neutral [72]. Data on the use of wrist supports and technique alterations is very limited, alterations in ergonomics to minimize fatigue may be key in reducing the impact of tremors during ophthalmic surgery.

### Lifestyle factors

The consumption of coffee is extremely common among doctors and even more prevalent in those working in surgical specialties [73]. While caffeine consumption temporarily improves alertness and cognitive functioning, it also adversely affects the severity of an individual’s physiologic tremor. Urso-Baiarda et al. double-blind crossover study observing end-to-end vessel anastomosis reported that caffeine had a deleterious effect on performance [38]. Another trial of vitreoretinal surgeons noted deleterious effects on Eyesi Surgical Simulator performance following lose-dose caffeine consumption [23]. Evidence on whether caffeine consumption impacts on surgical performance is currently limited, it is still recommended that caffeine intake immediately prior to surgery should be avoided.

Literature on the impact of diet on physiological tremors is not available to the best of our knowledge, however following a Mediterranean diet has been reported to be beneficial in the management of patients suffering from essential tremors [74]. An American study reported that both upper body only (i.e., using free weight/machine to train chest, shoulders, and arms) and aerobic exercise (i.e., running, cycling, stair climbing) for 30–60 min significantly increased microsurgical hand tremor immediately after exercise [75]. They found that the participants returned to their baseline tremor 2 h post-upper body only exercise and 4 h post-aerobic exercise. Physical activity has been demonstrated to increase circulating levels of catecholamines by 1.5 to >20 times (dependent on duration and intensity) within the body, leading to an increase in muscle activity and subsequent worsening of physiologic tremor [76]. Based on this finding avoiding exercise 2 to 4 h prior to performing ophthalmic microsurgery may be beneficial in minimizing the severity of a proceduralist’s tremor, however, it is worth noting that no studies have directly evaluated the impact of preceding exercise on surgical performance in terms of metrics or procedural outcomes. In the case of pathological tremors resistance training has demonstrated benefit in reducing the tremor amplitude of individual’s suffering from essential tremors [77–79]. Similarly, active and passive lower body cycling have demonstrated an improvement in Parkinson’s disease patients [80].

Recent times have seen increasing focus on yoga, mindfulness, and deep breathing exercises as potential tremor reduction strategies in microsurgery. These activities have demonstrated efficacy in reducing stress and improving general well-being [81, 82]. A study of 50 surgical residents found an objective reducing in hand tremor in participants when assessing their tremors prior to and after pranayama yoga [83]. Pranyama yoga involves four main principles: a stepwise reduction in breathing rate, aiming for 1:2 ratio of length of inspiration vs. expiration, holding ones breathing for twice as long as expiration, and focusing on the noise produced by air moving through the glottis. This paper by La Padula et al. is the first of its kind demonstrating tremor reduction following yoga/deep breathing exercises.

Alcohol consumption has been reported to be beneficial in the transient reduction of tremors in patients suffering from essential tremors (ET) [84]. Similarly, Lakie et al. found that alcohol consumption caused a significant transient reduction in physiological tremor at the wrist [85]. While alcohol consumption does demonstrate improvement in physiological tremor, its consumption immediately prior to and the day before surgery has been found to be detrimental to surgical performance [86]. Furthermore, Gallagher et al. reported that excessive alcohol consumption the night prior to operating resulted in a decreased surgical performance until at least 4 pm the following day [87]. These findings suggest that though alcohol may be beneficial in reducing the magnitude of an operator’s physiologic tremor, it is detrimental to overall surgical performance and as such should be avoided the day prior and immediately before surgery.

While literature is limited, there is data suggesting that smoking cigarettes may acutely worsen an individual’s tremor dating back to the 1970s [88]. Following this (in 1983), Shiffman et al. reported that smoking two cigarettes doubled participant’s tremor scores [89]. Another American study found that the smoking cohort had a greater tremor score when assessing both static and kinetic tremors in comparison with their non-smoking counterparts. The findings from these studies tend to suggest that smoking may be detrimental to surgeon hand tremor and thus performance, however, no study directly evaluating this was identified from the review of literature.

Broadly, recreational drugs such as cocaine, methamphetamines, ecstasy, and cannabis are strictly prohibited for use by surgeons. A review of literature did not identify any studies evaluating the use of these illicit substances and the impact upon surgical performance. In the case of methamphetamines and ecstasy, one study found that stimulant users self-reported negative impacts on fine hand control, in addition to worsened tremors [90]. Interestingly cannabidiol has been demonstrated to reduce anxiety and tremor amplitude [91]. The use of cannabis has also been trialed as a tremor-reducing strategy in pathologic tremors (e.g., Parkinson’s disease, multiple sclerosis), thus far demonstrating no benefit [92–94]. Flavel et al. explored the impact of these substances on resting and active tremors of participants using an accelerometer [95]. They found that during movement, tremor amplitude was significantly increased in ecstasy users compared to non-drug users. Interestingly, cannabis or amphetamine-like drugs were not noted to worsen tremors. In contrast, Bauer found that participants with cocaine dependence had greater hand tremors than their non-dependent counterparts [96]. While research on the impact of recreational substances on physiologic tremor amplitude is inconclusive, it is unlikely that significant literature exploring this topic further would be easily attainable due to ethical considerations.

In addition to this, an attempted review of literature to evaluate the impact of underlying health conditions (e.g., stroke or muscle atrophy) on surgeon tremor did not yield any significant literature. Furthermore, there does not appear to be any data evaluating the impact of therapies owing to specific pathologies and operator tremor.

## Limitations

One of the limitations of this review is the limited quality of published literature included. The majority of studies identified were Level IV and V evidence; thereby the strength of conclusions made have limited validity. The reasons for most studies being assigned lower levels of evidence include limited number of participants, studies with solely non-surgeon participants, subjects were phantom models or animals, and poor study design.

Another limitation of this review is a majority of studies originated from America. While the training of ophthalmologists and surgeons in America is likely comparable to countries such as the United Kingdom and Australia, the training of specialists in regions such as Asia and South America may be different. It is currently unclear the impact geographic training locations have on surgical performance and if the narrow geographic profile of countries included in this study may skew the findings noted.

A considerable proportion of the literature included is based on specialties other than ophthalmology. Although specialties such as neurosurgery may have some similarities to surgical approaches and techniques used in ophthalmology, several of the studies included in the review were based on General Surgery and the transferability of these findings is limited. While findings suggest that tremor reduction improves surgical performance in ophthalmic surgery, no robust evidence is available with regards to directly examining the effect of intraoperative tremor on surgical outcomes in live patients at this point in time.

Another limitation to this study was the significant variability in the methodology of tremor measurement. Strategies used to evaluate operator tremors included subjective reporting, positional sensors, software within surgical simulators, and motion tracking software. At present, there is no clear consensus as to which method is optimal for use in future studies. Furthermore, there is no data clearly identifying which specific parameters are the most robust indicators of measuring tremor severity (e.g., deviation from target point or maximum error while undertaking a specific surgical maneuver). Given the lack of consensus, exploring which methodology of tremor measurement and detection is most accurate warrants further exploration. This would allow for the design of more robust and consistent projects in the future.

## Conclusion

Ophthalmologists are required to operate at the peak of their physiological limits and have impeccable control of their physiologic tremor due to the precision and dexterity required to undertake eye surgery. Operating in these conditions is extremely challenging and leaves little margin for error. In recent times there has been increasing research to suppress a microsurgeon’s tremor. Robotic technology, new instruments, exoskeletons, technique modifications, and lifestyle factors have all demonstrated the potential to assist in overcoming tremors in ophthalmology. Presently, the main barrier to the incorporation of such technologies into widespread commercial use is the lack of published data on success and safety in human subjects. However, with continued development, the incorporation of these strategies into clinical practice is promising.

## Supplementary Information

Below is the link to the electronic supplementary material.

Supplementary file1 (DOCX 27 KB)
