# Supplementary material for: Overcoming the impact of physiologic tremors in ophthalmology
Source: Graefes Arch Clin Exp Ophthalmol. 2022 Jul 5;260(12):3723–36. doi: 10.1007/s00417-022-05718-2 (PMC9666294; doi:10.1007/s00417-022-05718-2)
Supplement: Supplementary file 1 — Supplementary file1 (DOCX 27 KB) [file 417_2022_5718_MOESM1_ESM.docx]

Online Resource s1: Non-ophthalmology based studies reporting the impact of physiologic tremors on surgical metrics (**This Table will be a Supplementary Table (1)**

| **Author** | **Strategy to reduce tremors** | **Surgical metric** | **Improved performance** | **Specialty** | **Evidence Quality** | **Year** | **Country** | **(n)** |
| --- | --- | --- | --- | --- | --- | --- | --- | --- |
| **Chandra et al.[10]** | Muscle fatigue | Accuracy of simulated laparoscopic surgical activities | Yes (lower fatigue) | General Surgery | III | 2014 | India | 8 |
| **Jensen et al.[27]** | Muscle cooling garment | Tremor amplitude, time to fatigue and suture time | Yes* | Surgery | III | 2016 | America | 19 |
| **Feng et al.^17^** | Robotic ENT (Ear, Nose, and Throat) Microsurgery System (REMS) | MTS, TTC and participant survey of microvascular anastomosis of a chicken ischiatic artery | Yes | Ear, Nose and Throat Surgery | III | 2017 | America | 7 |
| **Deshpande et al.[28]** | Computer-assisted laser microsurgery (CALM) system | Participant survey of usability, controllability and efficiency during TLM tasks on ex vivo pig larynxes | Yes | Ear, Nose and Throat Surgery | III | 2018 | Italy | 57 |
| **Willems et al.[29]** | Da Vinci surgical system | Objective Structured Assessment of Technical Skills score, TTC and comfort rating on anastomoses of artificial microvessels | Yes (complex exposures only) | Orthopaedic Surgery | IV | 2016 | America | 2 |
| **Mitsuishi et al.[30]** | Robotic surgical system | TTC, positioning and accuracy during tracing | Yes | Neurosurgery | IV | 2012 | Japan | 5 |
| **Prasad et al.[31]** | Zeus surgical system | Accuracy during simulated microsurgical techniques | No | Surgery | III | 2004 | America | 15 |
| **Garcia-Ruiz et al.[32]** | Zeus surgical system | TTC and suturing precision | No | General Surgery | III | 1998 | America | 20 |
| **Moorthy et al.[33]** | Da Vinci Surgical System | TTC and distance travelled | Yes | General Surgery | III | 2004 | United Kingdom | 10 |
| **Krapohl et al.[34]** | Robotic Surgical System | Attempts to grasp foreign body and time for holding catheter in vessel | Yes | Plastic Surgery | IV | 2001 | Germany | NS |
| **Moore et al.[35]** | Da Vinci surgical system | Accuracy under stress on lab based surgical task | Yes | General Surgery | IV | 2015 | United Kingdom | 32 |
| **Choi et al.[36]** | Active handheld micromanipulator | Accuracy while line tracing | Yes | Microsurgery | IV | 2007 | America | 7 |
| **Chauhan et al.[37]** | Robotic microsurgical forceps | Motion tracking error, device accuracy, completion time | Yes | Ear, Nose and Throat Surgery | IV | 2019 | Italy | 10 |
| **Urso-Baiarda et al.[38]** | Caffeine consumption 30-60 minutes | Global rating scale score | Yes | Microsurgery | III | 2007 | United Kingdom | 22 |
| **Goto et al.[39]** | Intelligent Armrest ('EXPERT') | Surgeon fatigue, difficulty performing procedure and complications | Yes | Neurosurgery | IV | 2013 | Japan | 6 |
| **Basaran et al.[40]** | Fatigue and sleep deprivation | Error score, global rating scale score and autopsy score performances | Yes | Plastic Surgery | III | 2015 | Turkey | 1 |
